# MicroRNA-194-5p Attenuates Doxorubicin-Induced Cardiomyocyte Apoptosis and Endoplasmic Reticulum Stress by Targeting P21-Activated Kinase 2

**DOI:** 10.3389/fcvm.2022.815916

**Published:** 2022-03-07

**Authors:** Hongge Fa, Dandan Xiao, Wenguang Chang, Lin Ding, Lanting Yang, Yu Wang, Mengyu Wang, Jianxun Wang

**Affiliations:** ^1^School of Basic Medicine, Qingdao University, Qingdao, China; ^2^Qingdao Women and Children’s Hospital, Qingdao University, Qingdao, China; ^3^Institute for Translational Medicine, Qingdao University, Qingdao, China

**Keywords:** doxorubicin, cardiotoxicity, miR-194-5p, ER stress, apoptosis

## Abstract

**Objective:**

Many studies have reported that microRNAs (miRs) are involved in the regulation of doxorubicin (DOX)-induced cardiotoxicity. MiR-194-5p has been reported significantly upregulated in patients with myocardial infarction; however, its role in myocardial diseases is still unclear. Various stimuluses can trigger the endoplasmic reticulum (ER) stress and it may activate the apoptosis signals eventually. This study aims to explore the regulatory role of miR-194-5p in DOX-induced ER stress and cardiomyocyte apoptosis.

**Methods:**

H9c2 was treated with 2 μM DOX to induce apoptosis, which is to stimulate the DOX-induced cardiotoxicity model. The expression of miR-194-5p was detected by quantitative real-time PCR (qRT-PCR); the interaction between miR-194-5p and P21-activated kinase 2 (PAK2) was tested by dual luciferase reporter assay; terminal deoxynucleotidyl transferase dUTP nick-end labeling (TUNEL) assay and caspase-3/7 activity were used to assess apoptosis; trypan blue staining was applied to measure cell death; Western blotting was performed to detect protein expressions; and ER-related factors splicing X-box binding protein 1 (XBP1s) was detected by polyacrylamide gel electrophoresis and immunofluorescence to verify the activation of ER stress.

**Results:**

MiR-194-5p was upregulated in cardiomyocytes and mouse heart tissue with DOX treatment, while the protein level of PAK2 was downregulated. PAK2 was predicted as the target of miR-194-5p; hence, dual luciferase reporter assay indicated that miR-194-5p directly interacted with PAK2 and inhibited its expression. TUNEL assay, caspase-3/7 activity test, and trypan blue stain results showed that either inhibition of miR-194-5p or overexpression of PAK2 reduced DOX-induced cardiomyocyte apoptosis. Silencing of miR-194-5p also improved DOX-induced cardiac dysfunction. In addition, DOX could induce ER stress in H9c2, which led to XBP1 and caspase-12 activation. The expression level of XBP1s with DOX treatment increased first then decreased. Overexpression of XBP1s suppressed DOX-induced caspase-3/7 activity elevation as well as the expression of cleaved caspase-12, which protected cardiomyocyte from apoptosis. Additionally, the activation of XBP1s was regulated by miR-194-5p and PAK2.

**Conclusion:**

Our findings revealed that silencing miR-194-5p could alleviate DOX-induced cardiotoxicity *via* PAK2 and XBP1s *in vitro* and *in vivo*. Thus, the novel miR-194-5p/PAK2/XBP1s axis might be the potential prevention/treatment targets for cancer patients receiving DOX treatment.

## Introduction

Doxorubicin (DOX) is a broad-spectrum antitumor drug that can be used to treat a variety of cancers. However, the clinical utility of DOX is confined due to its cumulative cardiotoxicity (1, 2). In the past decades, the mechanisms of DOX-induced cardiotoxicity have been extensively studied, mainly including accumulation of reactive oxygen species (ROS), mitochondrial dysfunction, endoplasmic reticulum (ER) stress, and disturbance of calcium homeostasis (3–8). However, the exact mechanism underlying DOX cardiotoxicity has not been fully discovered. In addition, the aberrant apoptosis caused cardiomyocytes number decrease is the predominant cellular event in DOX-induced cardiomyopathy, which was confirmed by morphological changes and terminal deoxinucleotidyl transferase dUTP nick-end labeling (TUNEL) assay (9–11). Therefore, to further explore the mechanisms of DOX-induced cardiomyocytes apoptosis will help minimize its adverse effects and benefit the clinical application.

MicroRNA (miR, miRNA) is a type of non-coding RNA with a length of approximately 22 nucleotides, and they exert their functions by degrading target mRNAs and inhibiting protein expressions, therefore, participate in various biological processes, such as proliferation, migration, differentiation, and cell death (12, 13). Many studies have reported that miRNAs play important roles in the DOX-induced cardiotoxicity (14–17). Recently, it has been reported that miR-194 is upregulated in the serum of patients with myocardial infarction and is closely correlated with impaired cardiac function (18). In addition, the expression level of circulating exosomal miR-194 was also upregulated in patients with obese cardiomyopathy, which was closely related to the mitochondrial activity and cardiac function (19). However, the role of miR-194 in DOX-induced cardiotoxicity is unclear.

P21-activated kinase 2 (PAK2), a Rac1/Cdc42 activated signaling effector, belongs to the PAK family of serine/threonine kinases (20). The antiapoptotic effect of PAK2 has been demonstrated in multiple cancer studies (21–23). Recently, PAK2 has been reported to exert cardioprotective role by improving ER function through the inositol-requiring enzyme 1 (IRE1)/X-box binding protein 1 (XBP1)-dependent pathway (24). In cardiomyocytes hypoxia and reoxygenation model, the decrease of PAK2 is associated with ER stress, oxidative stress, calcium overload, caspase-12 (cas-12) activation, and apoptosis (25). Activation of 5′ AMP-activated protein kinase (AMPK)-p21-activated kinase 2 (PAK2) signaling attenuated ER stress and myocardial apoptosis induced by ischemia/reperfusion injury (26). Nonetheless, the role of PAK2 in DOX-induced cardiotoxicity has not been elucidated.

It has been reported that ER stress is involved in DOX-induced cardiotoxicity (27, 28). When the ER is under stress that cannot afford the excessive unfolded proteins to be processed, the unfolded protein response (UPR) is triggered to restore the ER homeostasis (29, 30). Severe or prolonged ER stress will switch the cells from adaptive phase to apoptosis. XBP1 is the key transcription factor in the IRE pathway in response to UPR. During UPR, XBP1 is activated and its mRNA is cleaved to form the splicing XBP1 (XBP1s) (31). XBP1s can bind to ER stress response elements in promoters of many UPR target genes, therefore help to fold and degrade proteins, promoting ER adaption and cytoprotection (32, 33). Studies reported that XBP1s also plays a key role in cardiovascular disease. A recent study showed that XBP1s modulates vascular endothelial growth factor-mediated cardiac angiogenesis and contributes to the development of adaptive hypertrophy (34). Similarly, in the transgenic mouse model, overexpression of XBP1s showed protective effect on reperfusion injury (35). However, the role of XBP1 in DOX-induced cardiotoxicity needs further study.

In this study, we reported that the expression of miR-194-5p increased in DOX-induced cardiomyocytes and mouse heart tissue and was involved in the regulation of DOX-induced cardiotoxicity by targeting PAK2. Inhibition of miR-194-5p attenuated DOX-induced apoptosis, and PAK2 showed important role in maintaining endoplasmic reticulum homeostasis to exert cardioprotective effects *via* the key transcription factor-XBP1. The present results revealed the regulatory role of miR-194-5p/PAK2/XBP1s axis in DOX-induced cardiotoxicity and provided a theoretical basis for the development of therapeutic targets.

## Materials and Methods

### Animal Experiments

A 8-week old male C57BL/6J mice were randomly divided into the 4 groups: the control group, the DOX treatment group, the DOX and antagomir negative control group, and the DOX and miR-194-5p antagomir group. All the mice were housed on a 12-h light/12-h dark cycle in a pathogen-free environment and allowed *ad libitum* access to food and water. Adenovirus-harbored miR-194-5p antagomir (5 × 10^10^ vector genomes) was synthesized by Hanbio Corporation Ltd. (Shanghai, China). The animals in the antagomir group and its negative control (NC) group were injected *via* tail vein with miR-194-5p antagomir 50 μl or same dosage of antagomir NC. On day 7, the experimental groups (DOX group, DOX + antagomir NC group, and DOX + miR-194-5p antagomir group) were intraperitoneally injected with DOX hydrochloride 15 mg/kg once. Same dose of normal saline was injected to the control group. Cardiac function was tested 1 week after DOX administration and mice were euthanized after *in vivo* evaluations of cardiac function. Then, hearts were rapidly excised and immediately cut into two parts. One part was snap-frozen in liquid nitrogen and the remaining part was fixed in 4% polyformaldehyde solution and embedded in paraffin. All the procedures involving animals were reviewed and approved by the Institutional Animal Care and Use Committee of Qingdao University Medical College.

### Cell Culture and Treatment

H9c2 cells (rat cardiomyocytes) were purchased from the Shanghai Institutes for Biological Sciences (Shanghai, China), which were cultured in Dulbecco’s Modified Eagle’s Medium (DMEM) (Gibco; Thermo Fisher Scientific, Waltham, MA, United States) supplemented with 10% fetal bovine serum (FBS), 100 U/ml penicillin, 100 μg/ml streptomycin, and 110 mg/l sodium pyruvate at 37°C in a humidified atmosphere containing 5% CO_2_. The cells were treated with 2 μM or 0.2 μM DOX (Aladdin., Shanghai, China) at the indicated times.

### Cell Transfection

H9c2 cells were transfected with the Lipofectamine 3000 Transfection Reagent when they reached approximately 70% confluence for 24 h according to the manufacturer’s instructions. PAK2 and XBP1s were cloned into the pcDNA3.1 expression and synthesized by Tsingke (Beijing, China). The empty vector of pcDNA3.1 and scramble control were used as negative controls for overexpression and small interfering RNA (siRNA), respectively. MiR-mimic, miR-inhibitor, and si_PAK2 were purchased from Shanghai GenePharma (Shanghai, China). Their sequences are shown in Table 1.

**TABLE 1:** The sequences of synthesized mimic, inhibitor, small-interfering RNA (siRNA).

Gene	Sequence
miR-194-5p mimic	F: UGUAACAGCAACUCCAUGUGGA
	R: CACAUGGAGUUGCUGUUACAUU
mimic-NC	F: UUCUCCGAACGUGUCACGUTT
	R: ACGUGACACGUUCGGAGAATT
miR-194-5p inhibitor	5′-UCCACAUGGAGUUGCUGUUACA-3′
Negative control	5′-CAGUACUUUUGUGUAGUACAA-3′
Si_PAK2	5′-GGGAAUGGAAGGCUCAGUUTT-3′
Scramble control	5′-UUCUCCGAACGUGUCACGUTT-3′

### Quantitative Real-Time PCR

Total RNA obtained from the H9c2 cells or left ventricle tissue was extracted using Trizol reagent. RNA was reverse transcribed with HiScript III RT SuperMix for qPCR (+ gDNA WIper) reverse transcription kit (Vazyme, Nanjing, China) for mRNA levels testing. Stem-loop quantitative real-time PCR (qRT-PCR) for mature miRNAs was performed as previously described (36) with miRNA 1st Strand cDNA Synthesis Kit (by stem-loop) (Vazyme, Nanjing, China) for miRNA levels testing. The miR-194-5p stem-loop primer sequence: 5′-GT CGTATCCAGTGCAGGGTCCGAGGTATTCGCACTGGATAC GACTCCACA-3′. According to the manufacturer’s instructions, the cDNA was mixed with the corresponding fluorescent dye SYBR, and the test was carried out in the CFX96 real-time PCR system (Bio-Rad, Hercules, CA, United States). The results were put into the 2−^ΔΔ^CT formula for calculation. MiR-194-5p expression was normalized to that of U6, while XBP1s mRNA level was normalized to that of glyceraldehyde 3-phosphate dehydrogenase (GAPDH). The primers are shown in Table 2.

**TABLE 2:** Real-time quantitative PCR (qRT-PCR) primers used in this study.

Gene	Sequence
U6	F: ATTGGAACGATACAGAGAAGATT
	R: GGAACGCTTCACGAATTTG
miR-194-5p	F: CGCGTGTAACAGCAACTCCA
	R: AGTGCAGGGTCCGAGGTATT
GAPDH	F: GCCCATCACCATCTTCCAGGAG
	R: GAAGGGGCGGAGATGATGAC
XBP1s	F: TGAGAACCAGGAGTTAAG
	R: CCTGCACCTGCTGCGGAC

### Cell Apoptosis Assay

The sterile slides were placed in the 24-well plate and then the H9c2 cells were planted on top of the slides. After transfection and treatment, 4% paraformaldehyde added to fix the cells for at least 1 h in room temperature. Cell apoptosis was characterized *via* a TUNEL assay using the TUNEL Apoptosis Detection Kit (YEASEN, Shanghai, China) according to the manufacturer’s instructions. The samples were mounted with mounting medium containing 4′,6′-diamidino-2-phenylindole (DAPI) (Vector Laboratories, Burlingame, CA, United States) to stain nuclei. The stained-glass slides were observed and photographed under a fluorescence microscope. The percentage of the apoptotic nuclei was calculated by the number of apoptotic cells/the number of total nuclei. We randomly measured 150 cells from each experiment to calculate the apoptotic rate. Caspase-3/7 activity assay was performed using the Caspase 3/7 Activity Assay Kit (Meilunbio, Dalian, China) according to the manufacturer’s instructions. Masson’s trichrome staining was performed using the staining kit (Solarbio, Beijing, China) following the manufacturer’s instructions.

### Trypan Blue Stain

Cell death rate was measured by trypan blue stain (Solarbio, Beijing, China). The supernatant and adherent cells were collected. The cell was prepared and stained by trypan blue according to the manufacturer’s instructions. The percentage of the cell death was calculated by the number of trypan blue positive cells/the number of total cells, which were counted under the microscope.

### Western Blot Analysis

Total protein was extracted from H9c2 cells or mouse left ventricle tissue by the radio immunoprecipitation assay (RIPA) Lysis Buffer (Solarbio, Beijing, China) according to the manufacturer’s instructions. Proteins were separated by electrophoresis on the sodium dodecyl sulfate-polyacrylamide gel electrophoresis (SDS-PAGE) (10–12% polyacrylamide gels) and then transferred to polyvinylidene fluoride (PVDF) membranes. Subsequently, the PVDF membranes were blocked in 5% non-fat milk for 2 h and then incubated overnight at 4°C with anti-PAK2 (1:1,000, Cell Signaling Technology, Danvers, MA, United States), or anti-XBP1s (1:1,000, Cell Signaling Technology, Danvers, MA, United States), or anti-β-actin (1:2,000, Santa Cruz Biotechnology, Dallas, TX, United States), or anti-GAPDH (1:100,000, ABclonal, Wuhan, China), or anti-cas-12 (1:2,000, Abcam, United States) primary antibodies after washing with TBS-Tween 20 (TBST) three times, 10 min each time. Horseradish peroxidase (HRP)-conjugated secondary antibodies were incubated at room temperature for 1 h, then washed with TBST three times, 10 min each time. Membranes were visualized using enhanced chemiluminescence. Protein expression was quantified using ImageJ, and β-actin or GAPDH was used as the internal control.

### Dual-Luciferase Reporter Gene Assay

The wild-type (WT) and mutated-type (MT) PAK2 fragments of the miR-194-5p binding region were, respectively, inserted into the pGL3 vector immediately downstream of the stop codon of the luciferase gene, to synthesize the reporter gene plasmid (Tsingke, Beijing, China). A luciferase activity assay was performed as described previously (37). Briefly, phRL-TK reporter plasmid and miR-194-5p mimic (or mimic-NC) were cotransfected into HEK-293 cells, which were seeded in 48-well plates. The cells were collected and lysed after 24 h, then the firefly and Renilla luciferase activities were detected by the Dual–Luciferase Reporter Assay System (Promega, Madison, WI, United States). Firefly luciferase activities were normalized to Renilla luciferase activity.

### Polymerase Chain Reaction Product Polyacrylamide Gel Electrophoresis

The extracted RNA was first reverse transcribed into cDNA with HiScript III RT SuperMix for qPCR (+ gDNA wiper) (Vazyme, Nanjing, China). The cDNA was amplified by PCR with Gold Mix rapid PCR enzyme (Tsingke, Beijing, China). About 10% of polyacrylamide gel (per 10 ml: 30% acrylamide 3.33 ml, 10X TBE 1 ml, ddH_2_O 5.614 ml, N,N,N′,N′-Tetramethylethylenediamine (TEMED) 5 μl, 10% ammonium persulfate (APS) 50 μl) were prepared. Electrophoresis was performed in 1 × TBE solution and the PCR products were separated. Then gel was stained in Gelred non-toxic nucleic acid dye in the dark (dye: water = 1:10,000 ratio) for 30 min and visualized using chemiluminescence.

### Immunofluorescence

Cells were planted and fixed in the same manner as TUNEL assay. About 0.5% Triton X-100 was used for cell permeability treatment for 30 min. After discarded Triton X-100, cells were rinsed with phosphate-buffered saline (PBS) for three times, 5 min each time. Blocked with goat serum for 1 h, then washed with PBS for three times, 5 min each time. Added primary antibody and incubated overnight at 4°C, then washed with PBS. Fluorescent secondary antibody was added and incubated in dark for 1 h. After washing with PBS, slides were mounted with DAPI to stain nuclei. The slides were observed and photographed using an inverted two-photon laser confocal microscope.

### Echocardiographic Assessment

Generally, mice were mildly anesthetized with intraperitoneal injection of 4% chloral hydrate 0.1 ml/10 g, and the hair over the chest region was removed. The mice were then placed in a supine position and transthoracic echocardiography was performed using a VINNO 6 Lab system (VINNO, Suzhou, China). Two-dimensional guided M-mode tracings were recorded in parasternal long and short axis views at the level of the papillary muscles. Left ventricular ejection fraction (EF) and fractional shortening (FS) were recorded by the system. All the measurements were obtained for greater than three beats and averaged.

### Statistical Analysis

The experimental data were analyzed using GraphPad Prism version 5 software and the data were presented as mean ± SD. *T*-test was used to compare the data between the two groups. One-way ANOVA was used to compare the mean values of multiple groups. Tukey’s *post hoc* test was used for pairwise comparison between the multiple groups. All the experiments were repeated three times and *p* < 0.05 was indicated as statistically significant.

## Results

### MicroRNA-194-5p Participated in Doxorubicin-Induced Cardiomyocyte Apoptosis

**FIGURE 1:**
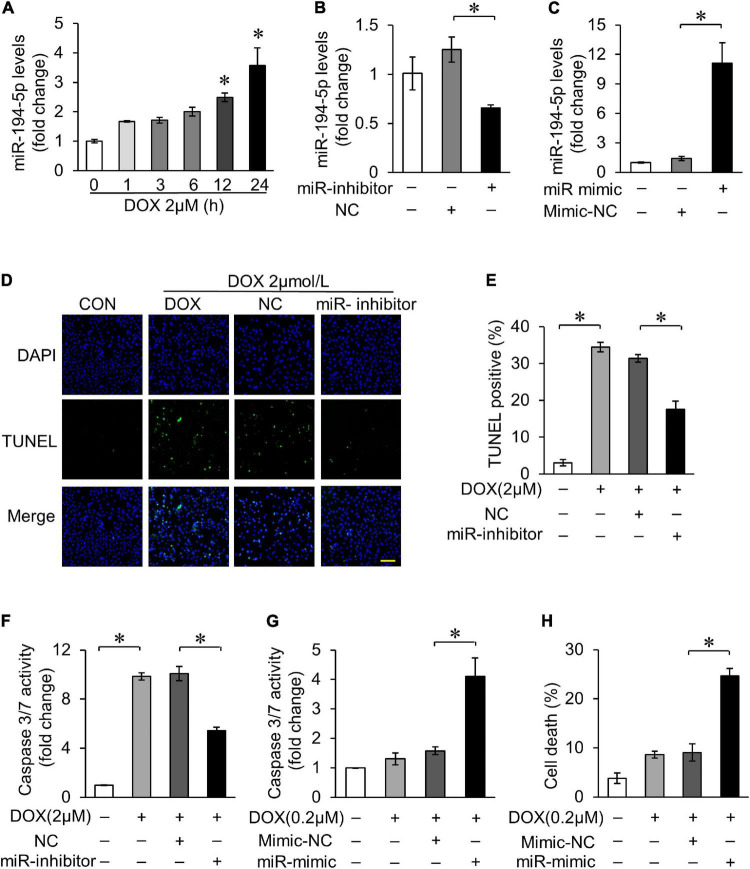
MiR-194-5p participated in doxorubicin (DOX)-induced cardiomyocyte apoptosis. **(A)** H9c2 cells were treated with 2 μM DOX for the indicated times. The expression levels of miR-194-5p were measured by qRT-PCR. *p < 0.01 vs. control. **(B)** MiR-194-5p expression after transfection with miR-194-5p inhibitor for 24 h was measured by real-time quantitative PCR (qRT-PCR). **(C)** MiR-194-5p expression after transfection with miR-194-5p mimic for 24 h was measured by qRT-PCR. **(D–F)** Suppressed miR-194-5p expression with miR-194-5p inhibitor for 24 h and exposed cells to 2 μM DOX for 24 h. Apoptosis was detected by terminal deoxinucleotidyl transferase dUTP nick-end labeling (TUNEL) assay **(D)**. Green, TUNEL-positive nuclei; blue, 4,6-diamidino-2-phenylindole (DAPI)-stained nuclei; scale bar, 200 μm. Statistical analysis of TUNEL-positive cells **(E)** and caspase 3/7 activity **(F)** are shown. **(G,H)** Enhanced miR-194-5p expression with miR-194-5p mimic for 24 h and exposed cells to 0.2 μM DOX for 24 h. Caspase-3/7 activity **(G)** and cell death rate **(H)** are shown. All the experiments have been performed independently in triplicate, and the data were expressed as mean ± SD. *p < 0.01 as indicated.

We first investigated the sequences of miR-194-5p, and found that they are homologous in human, rat, and mouse according to miRBASE (miRBASE Sequence database-release 22.1). In order to explore the role of miR-194-5p in DOX-induced cardiotoxicity, rat myocardial cell line H9c2 was treated with 2 μM DOX to simulate the cell model of DOX-induced cardiotoxicity. With 2 μM DOX treatment, the expression of miR-194-5p increased in a time-dependent manner (Figure 1A). Transfection with miR-194-5p inhibitor could effectively suppress the expression of miR-194-5p (Figure 1B), while transfection with miR-194-5p mimic enhanced its expression (Figure 1C). Next, we further studied the potential role of miR-194-5p in DOX-induced cardiomyocytes apoptosis. When miR-194-5p expression was inhibited, DOX-induced apoptosis was significantly reduced on TUNEL assay (Figures 1D,E). In addition, inhibition of miR-194-5p attenuated DOX-induced caspase-3/7 activity elevation (Figure 1F). On the other hand, in order to demonstrate whether miR-194-5p participate in regulating the sensitivity of cardiomyocytes to DOX, low dose of DOX (0.2 μM) was used to treat cardiomyocytes. Under low DOX concentration stimulation, overexpression of miR-194-5p sensitized cardiomyocytes to cas-3/7 activity elevation (Figure 1G). Since apoptosis is the predominant cell death mode in DOX-induced cardiotoxicity, the detection of cell death rate can also reflect the degree of DOX-induced cardiotoxicity. Finally, increased cell death induced by low dose DOX was further aggravated by miR-194-5p mimic (Figure 1H). Taken together, miR-194-5p was upregulated in cardiomyocytes with DOX treatment, and inhibition of miR-194-5p could alleviate DOX-induced apoptosis.

### MicroRNA-194-5p Directly Targeted P21-Activated Kinase 2

**FIGURE 2:**
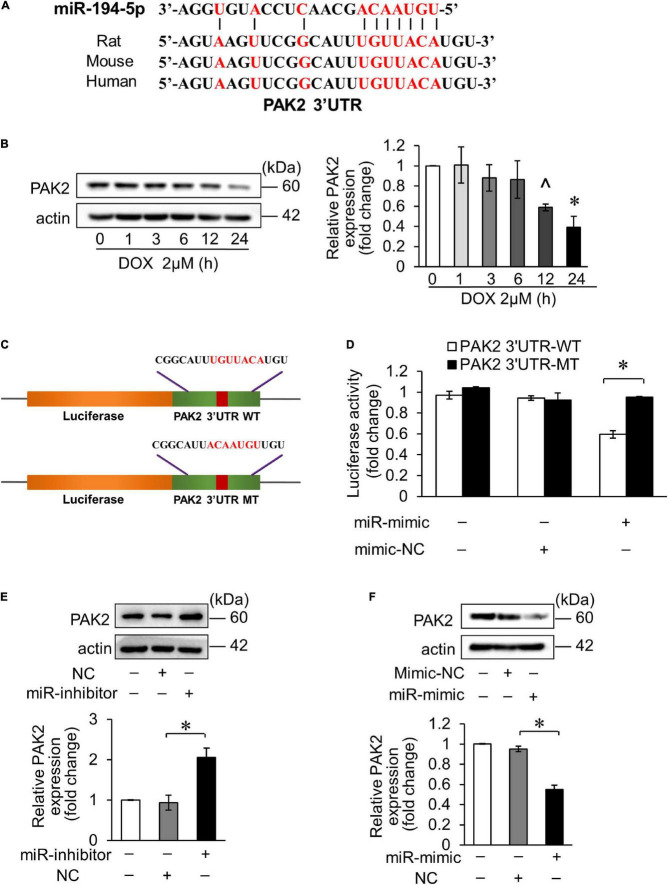
MiR-194-5p directly targeted PAK2. **(A)** Prediction of the PAK2 3′UTR potential binding site of miR-194-5p *via* bioinformatics program TargetScan. Potential complementary bases are shown in red. **(B)** H9c2 were treated with 2 μM DOX for the indicated times. The expression levels of PAK2 were detected by Western blots and the quantitative histogram was shown. ∧*p* < 0.05 vs. control. *p < 0.01 vs. control. **(C)** Schematic diagram of the reporter containing the putative miR-194-5p binding site in the PAK2 3′UTR region. WT, wild-type; MT, mutant. **(D)** Luciferase activity detected in HEK-293 cells transfected with miR-194-5p mimic or negative control along with luciferase reporter constructs, as indicated. **(E)** H9c2 was transfected with miR-194-5p inhibitor for 24 h. The expression levels of PAK2 were detected by Western blot and the quantitative histogram was shown. **(F)** H9c2 was transfected with miR-194-5p mimic for 24 h. The expression levels of PAK2 were detected by Western blot and the quantitative histogram was shown. All the experiments have been performed independently in triplicate, and the data were expressed as mean ± SD. *p < 0.01 as indicated.

It was predicted that miR-194-5p directly binds to PAK2 3′ untranslated region (UTR) region on the bioinformatics program TargetScan. Moreover, PAK2 has conserved binding sites for miR-194-5p (Figure 2A). Hence, we tested PAK2 expression level in DOX-treated H9c2, and the result showed that its expression level was significantly decreased 12 h after treatment (Figure 2B). Then, we speculated the regulatory effect of miR-194-5p on DOX-induced cardiomyocyte apoptosis achieved by targeting PAK2. To verify whether miR-194-5p directly binds to PAK2, we first constructed the luciferase plasmid containing the wild type of the predicted PAK2 3′UTR binding site (WT) or mutant binding site (MT) (Figure 2C). Dual luciferase reporter assay demonstrated that the fluorescence activity was inhibited when the WT plasmid was cotransfected with miR-194-5p mimic. The fluorescence activity remained unchanged when the MT plasmid was cotransfected with a miR-194-5p mimic, which indicated that miR-194-5p directly bound to PAK2 3′UTR region (Figure 2D). Next, we transfected miR-194-5p inhibitor and mimic into H9c2 cells to investigate their effects on PAK2 protein expression. MiR-194-5p inhibitor enhanced PAK2 expression (Figure 2E), while miR-194-5p mimic suppressed PAK2 expression (Figure 2F). These results indicated that miR-194-5p directly targeted PAK2 and negatively regulated its expression.

### P21-Activated Kinase 2 Attenuated Doxorubicin-Induced Cardiomyocyte Apoptosis

**FIGURE 3:**
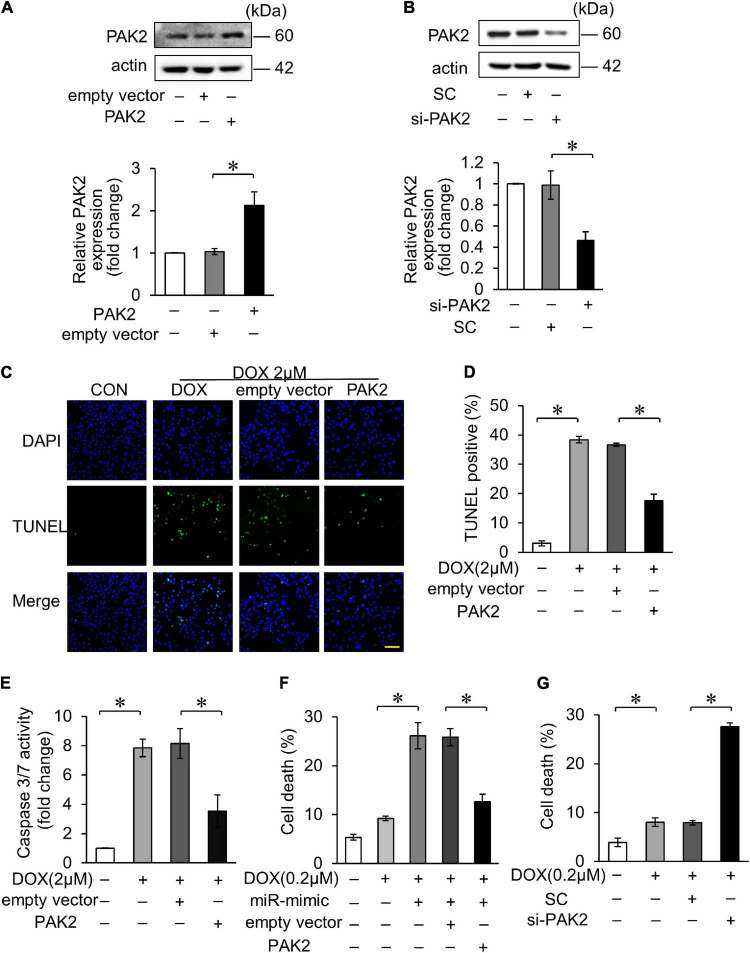
PAK2 attenuated DOX-induced cardiomyocyte apoptosis. **(A)** H9c2 was transfected with PAK2-overexpressing plasmid for 24 h. The expression levels of PAK2 were detected by Western blot and the quantitative histogram was shown. **(B)** H9c2 was transfected with PAK2 small-interfering RNA (siRNA) for 24 h. The expression levels of PAK2 were detected by Western blot and the quantitative histogram was shown. **(C–E)** Enhanced PAK2 expression with PAK2-overexpressing plasmid for 24 h and exposed to 2 μM DOX for 24 h. Apoptosis was detected by TUNEL assay **(C)**. Green, TUNEL-positive nuclei; blue, DAPI-stained nuclei; scale bar, 200 μm. Statistical analysis of TUNEL-positive cells **(D)** and caspase-3/7 activity **(E)** are shown. **(F)** H9c2 was cotransfected with miR-194-5p mimic and PAK2-overexpressing plasmid for 24 h and then exposed to 0.2 μM DOX for 24 h. Cell death rate was analyzed. **(G)** H9c2 was transfected with PAK2 siRNA for 24 h and exposed to 0.2 μM DOX for 24 h. Cell death rate was analyzed. All the experiments have been performed independently in triplicate, and the data were expressed as mean ± SD. *p < 0.01 as indicated.

We further investigated the role of PAK2 in DOX-induced cardiomyocytes apoptosis. The PAK2 plasmid was able to enhance its expression and si_PAK2 inhibited the expression (Figures 3A,B). Functionally, overexpression of PAK2 significantly decreased DOX-induced apoptosis (Figures 3C,D) and caspase-3/7 activity (Figure 3E). In addition, PAK2 overexpression abolished the effects of miR-194-5p on DOX-induced cell death (Figure 3F), indicating that PAK2 was the downstream target of miR-194-5p. Contrarily, cell death induced by 0.2 μM DOX was further increased with si_PAK2 (Figure 3G). The above findings indicated that the PAK2 could alleviate apoptosis in H9c2 cells exposed to DOX treatment.

### X-Box Binding Protein 1 Participated in Doxorubicin-Induced Cardiotoxicity

**FIGURE 4:**
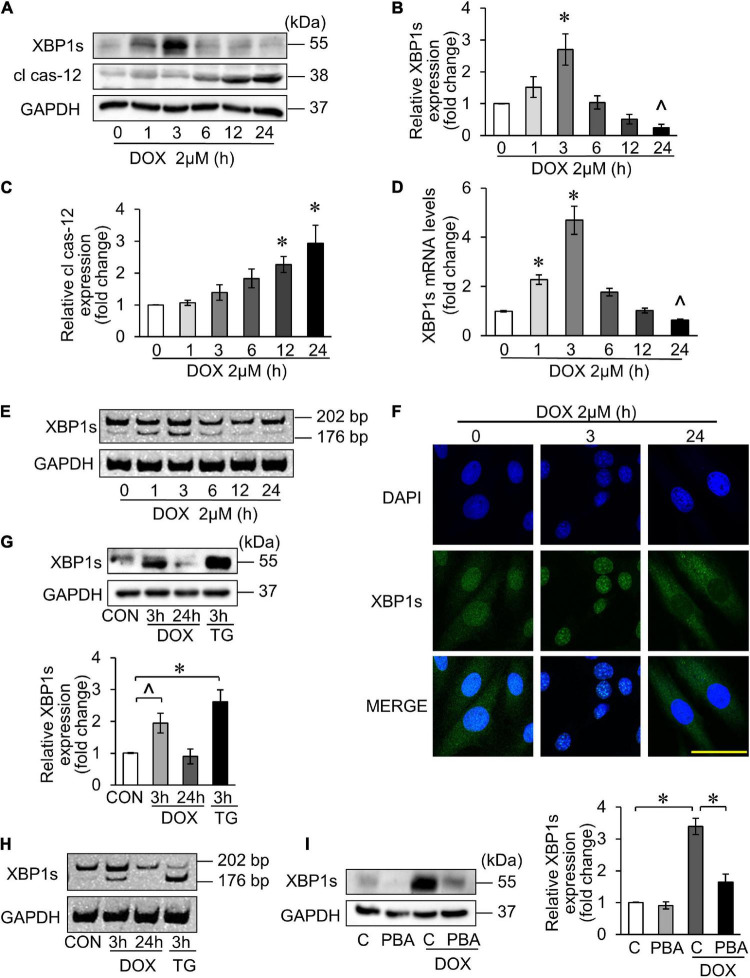
X-box binding protein 1 (XBP1) participated in DOX-induced cardiotoxicity. **(A–E)** H9c2 was treated with 2 μM DOX for the indicated times. The XBP1s levels were detected using Western blots **(A)** and the quantitative histogram was shown **(B,C)**, and also detected using qRT-PCR **(D)**. *∧p* < 0.05 vs. control. *p < 0.01 vs. control. Spliced bands of XBP1 were detected by polyacrylamide gel electrophoresis **(E)**. **(F)** H9c2 was treated with 2 μM DOX for 3 and 24 h. The localization of XBP1s in cells was detected by immunofluorescence experiment. Green, XBP1s; blue, DAPI-stained nuclei; scale bar, 50 μm. **(G,H)** H9c2 was treated with 2 μM DOX or 50 nM TG for the indicated times. The XBP1s levels were detected by Western blots and the quantitative histogram was shown **(G)**, and the spliced bands were detected by polyacrylamide gel electrophoresis **(H)**. TG, thapsigargin. **(I)** H9c2 was treated with 2 μM DOX for 3 h, which was pre-treated with 5 mM 4-PBA for 3 h. The XBP1s levels were detected by Western blots and the quantitative histogram was shown. All the experiments have been performed independently in triplicate, and the data were expressed as mean ± SD. ∧*p* < 0.05 vs. control. *p < 0.01 vs. control.

It has been reported that DOX-induced cardiotoxicity may activate multiple UPR pathways (28). The key transcription factor XBP1s is regulated by PAK2 in the heart (24). Therefore, we first explored the XBP1s expression in DOX-induced cardiotoxicity. Similarly, H9c2 was treated with 2 μM DOX for the indicated time, and the XBP1s expression reached peak at 3 h and decreased thereafter, which indicated the activation of the IRE/XBP1 pathway of UPR (Figures 4A,B).

Cas-12 as an indicator of ER-mediated apoptosis was investigated as well. The expression of its activated form—cleaved cas-12 (cl cas-12) was significantly increased 12 h onward under 2 μM DOX treatment (Figures 4A,C). Next, we detected the mRNA level of XBP1s, and the result showed the same trend with its protein expression levels (Figure 4D). When XBP1 was activated, XBP1 mRNA was spliced and 26 bases were cut off to form the splicing XBP1, also known as its activated form (XBP1s). Thus, we measured the cDNA level after reverse transcription from total RNA. The results also showed that significant XBP1s band appeared at 3 h after DOX treatment (Figure 4E). It has been reported that the XBP1s can be translocated from cytoplasm to nucleus once activated (38), and this can be confirmed by immunofluorescence experiments (Figure 4F). Next, Thapsigargin, an ER stress inducer, was used as the positive control to verify that DOX could trigger the UPR and activate the XBP1s (Figures 4G,H). The inhibition of the ER stress by 4-PBA inhibited the DOX-triggered XBP1s at 3 h (Figure 4I). Taken together, the UPR was involved in DOX-induced cardiotoxicity, in which XBP1 was activated. In addition, the XBP1s expression reached its peak at 3 h in DOX-treated H9c2, and then decreased.

### X-Box Binding Proteins 1 Attenuated Doxorubicin-Induced Cardiomyocyte Apoptosis

**FIGURE 5:**
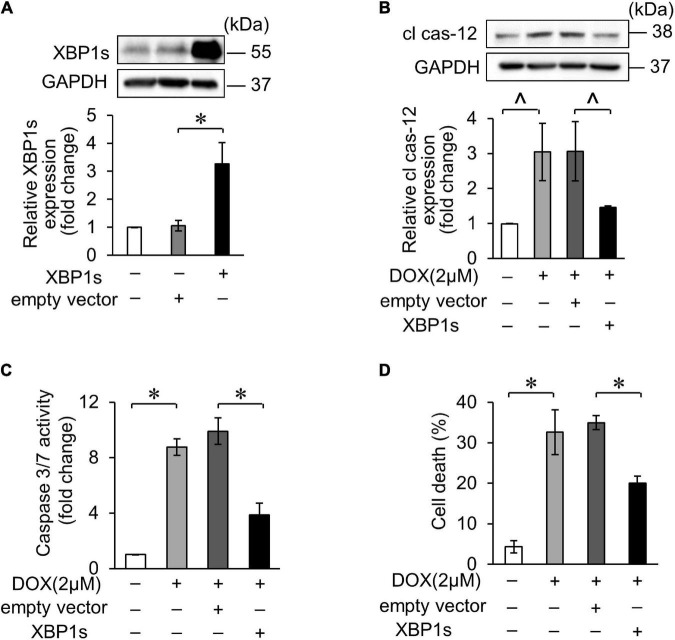
XBP1s attenuated DOX-induced cardiomyocyte endoplasmic reticulum (ER) stress and apoptosis. **(A)** H9c2 was transfected with XBP1s-overexpressing plasmid for 24 h. The expression levels of XBP1s were determined using Western blot and the quantitative histogram was shown. **(B–D)** Enhanced XBP1s expression in H9c2 cells with XBP1s-overexpressing plasmid for 24 h and exposed to 2 μM DOX for 24 h. The expression levels of cleaved caspase-12 were determined using Western blot and the quantitative histogram was shown **(B)**. Caspase-3/7 activity **(C)** and cell death rate **(D)** were analyzed. All the experiments have been performed independently in triplicate, and the data were expressed as mean ± SD. ∧*p* < 0.05 vs. control. *p < 0.01 vs. control.

Several studies have reported that XBP1s plays protective roles in the heart. In our study, we also confirmed the role of XBP1s in DOX-induced cardiotoxicity. The overexpression of XBP1s was verified by WB after transfection of XBP1s plasmid (Figure 5A). Cleaved caspase-12 expression increased in DOX-induced cardiomyocytes, indicating that the DOX-induced ER-related apoptosis, which decreased when XBP1s was overexpressed (Figure 5B). In addition, the overexpression of XBP1s significantly inhibited the DOX-induced increase in cas-3/7 activity (Figure 5C). Trypan blue stain assay showed the same result that the overexpression of XBP1s inhibited increased cell death rate induced by the DOX (Figure 5D). These results indicated that XBP1s could alleviate the ER-related apoptosis induced by the DOX and play the cardioprotective role.

**FIGURE 6:**
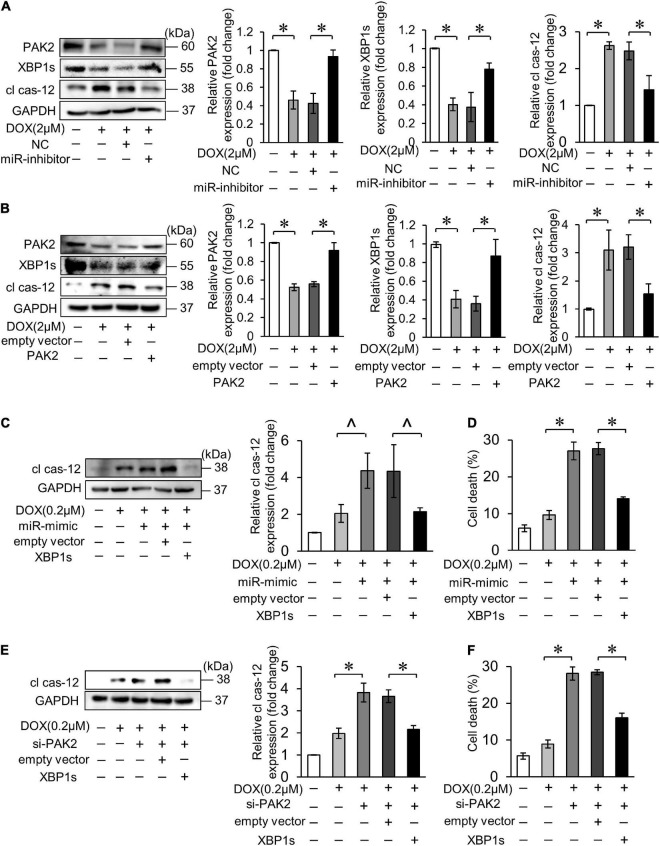
MiR-194-5p participated in DOX-induced ER stress and cardiomyocyte apoptosis through PAK2 and XBP1s. **(A)** Suppressed miR-194-5p expression with miR-194-5p inhibitor for 24 h and exposed to 2 μM DOX for 24 h. The expression levels of PAK2, XBP1s, and cleaved caspase-12 were detected by Western blot and the quantitative histogram was shown. **(B)** Enhanced PAK2 expression with PAK2-overexpressing plasmid for 24 h and exposed to 2 μM DOX for 24 h. The expression levels of PAK2, XBP1s, and cleaved caspase-12 were detected by Western blot and the quantitative histogram was shown. **(C,D)** H9c2 was cotransfected with miR-194-5p mimic and XBP1s-overexpressing plasmid for 24 h, then exposed to 0.2 μM DOX for 24 h. The expression levels of cleaved caspase-12 were detected by Western blot and the quantitative histogram was shown **(C)** and cell death rate was analyzed **(D)**. **(E,F)** H9c2 was co-transfected with PAK2 siRNA and XBP1s-overexpressing plasmid for 24 h, then exposed to 0.2 μM DOX for 24 h. The expression levels of cleaved caspase-12 were detected by Western blot and the quantitative histogram was shown **(E)** and cell death rate was analyzed **(F)**. All the experiments have been performed independently in triplicate, and the data were expressed as mean ± SD. ∧*p* < 0.05 vs. control. *p < 0.01 vs. control.

Activation of XBP1 has been shown to require the presence of PAK2 in cardiomyocytes. Next, we verified the relationship between miR-194-5p, PAK2 and XBP1s on the regulation of cardiomyocyte apoptosis. Firstly, inhibition of miR-194-5p could alleviate the downregulation of XBP1s expression and the upregulation of cl cas-12 expression levels under the DOX treatment (Figure 6A). Next, PAK2 restoration by transfection with its overexpression plasmid also reduced the downregulation of XBP1s expression and the upregulation of cl cas-12 expression levels (Figure 6B). When cotransfected, XBP1s partially eliminated miR-194-5p mimic caused elevation of cleaved cas-12 and cell death (Figures 6C,D). Similarly, XBP1s also partially eliminated si_PAK2 caused elevation of cleaved cas-12 level and cell death (Figures 6E,F). Thus, those data suggested that miR-194-5p and PAK2 regulated DOX-induced cardiomyocyte apoptosis *via* XBP1s.

### MicroRNA-194-5p Was Involved in Doxorubicin-Induced Cardiotoxicity *in vivo*

**FIGURE 7:**
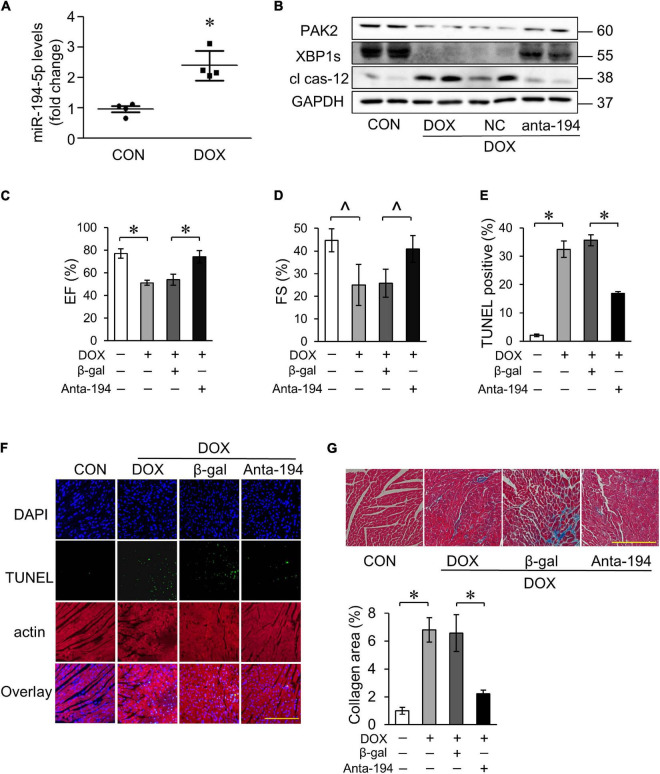
MiR-194-5p was involved in DOX-induced cardiotoxicity *in vivo*. **(A)** The expression levels of miR-194-5p in mice heart tissue were detected after DOX treatment by qRT-PCR. **(B)** Adenovirus-harbored anta-miR-194-5p was injected into the mice 1 week before DOX treatment. The expression levels of PAK2, XBP1s and cleaved caspase-12 were detected by Western blot. Echocardiographic analysis of left ventricular cardiac function in mice, EF **(C)** and FS **(D)** results are shown. EF, ejection fraction; FS, fractional shortening. Apoptosis was measured by TUNEL assay **(F)** and apoptotic rates were analyzed **(E)**. Green, TUNEL-positive nuclei; blue, DAPI (4,6-diamidino-2-phenylindole)-stained nuclei; scale bar, 500 μm. **(G)** Masson trichrome staining for collagen performed. scale bar, 200 μm. Anta-194, adenovirus-harbored miR-194-5p antagomir. *N* = 4, and the data were expressed as mean ± SD. ∧*p* < 0.05 vs. control. *p < 0.01 vs. control.

We further explored the role of miR-194-5p in the DOX-induced cardiotoxicity in the mouse model. We found that DOX treatment induced an increase in miR-194-5p expression levels in the heart (Figure 7A). Moreover, the protein expression levels of PAK2, XBP1s decreased, and cl cas-12 level increased (Figure 7B). Next, we validated the role of miR-194-5p *in vivo*. Injection with adenovirus-harbored miR-194-5p antagomir could reverse the expression of PAK2, XBP1s, and cl cas-12 induced by the DOX (Figure 7B). Furthermore, suppression of the miR-194-5p significantly improved cardiac function (Figures 7C,D), attenuated DOX-induced cardiomyocyte apoptosis (Figures 7E,F), and ameliorated myocardial fibrosis (Figure 7G). Taken together, our *in vivo* results showed a significant protective role of miR-194-5p antagomir in the DOX-induced cardiotoxicity.

## Discussion

Doxorubicin is the representative of anthracycline family, one of the most widely used effective antitumor drugs. However, DOX-induced cardiotoxicity is the major limiting factor for its application, and the cardiomyopathy may not be detected until years after the DOX completion. It has been reported that 10% of patients receiving DOX developed symptomatic cardiomyopathy within 15 years after the end of treatment (39). Studies over the years have revealed that oxidative stress and mitochondrial damage are the predominant mechanisms of DOX-induced cardiotoxicity. However, the simply use of antioxidants does not provide much protection against heart damage caused by DOX (40). This suggests that the DOX-induced cardiotoxicity may be the result of multiple mechanisms. In this study, we explored the molecular mechanisms involved in ER stress-related DOX cardiotoxicity, which provides a new strategy for the prevention and control of DOX-induced cardiotoxicity.

A growing number of studies have proposed miRNAs as potential targets for the DOX-induced cardiotoxicity. For example, in the DOX-induced cardiotoxicity, miR-15b-5p, miR-23a, miR-29b, and miR-146a have been proved to be related to mitochondrial damage; miR-30 family, miR-140-5p, and miR-451 are related to oxidative stress; miR-378 is associated with the ER stress and miR-320 is related to the microvascular density (17). We reported here that miR-194-5p participated in the DOX-induced cardiotoxicity and suppression of miR-194-5p alleviated the DOX-induced cardiomyocyte apoptosis.

In recent years, the role of ER stress in the DOX-induced cardiomyopathy gained attentions. Studies have shown that the DOX caused significant ER dilatation in human hearts (27). The effectors of ER stress were activated in the DOX-treated heart tissue, indicating that UPR was involved in regulating cardiomyocyte survival or death. Recent study has shown that PAK2 regulation of the protective ER function was through the IRE1/XBP1-dependent UPR pathway and this regulation was conferred by PAK2 inactivation of PP2A. Mice with PAK2 deletion showed defective response to ER stress, increasing cardiomyocyte damage (24). In our study, we demonstrated that PAK2 as the target gene of miR-194-5p exerted antiapoptotic effect in the DOX-induced cardiotoxicity.

In addition, the activation of the transcription factor XBP1 upregulates the expression of ER chaperone and ER associated degradation (ERAD) components to relieve ER stress and promotes cell survival (38). For example, XBP1^–/–^ livers showed increased apoptosis, and XBP1^–/–^ mouse embryos could not survive, while XBP1 transgenic reversed this embryonic lethality (41). In cardiovascular disease, cardiomyocyte-specific deletion of XBP1 aggravated cardiac dysfunction in ischemia-reperfusion injury, suggesting that XBP1s has a protective effect (35). The expression of XBP1s was decreased in the heart tissue of both human and rodents with heart failure, heart-specific XBP1 overexpression prevented the development of cardiac dysfunction, and XBP1s stimulated adaptive heart growth by activating mammalian target of rapamycin (mTOR) signal (42). In vascular smooth muscle cells, XBP1s promoted the repair of vascular injury or the formation of neointimal (43). These results indicated that XBP1s is involved in the regulation of cardiovascular disease and plays a protective role. Besides, other ER stress sensor, such as, binding immunoglobulin protein (BiP) was reported to bind to the IRE1 and protein kinase R-like endoplasmic reticulum kinase (PERK) *via* its nuclei binding domain (44); ER stress with prolonged activation of the UPR-initiated apoptotic cell death *via* the upregulation of C/EBP-homologus protein (CHOP). Previous studies showed that the DOX treatment increased CHOP, BiP, and cas-12 activation to initiated apoptosis (45, 46). In the case of DOX-induced cardiotoxicity, a study also revealed that XBP1s significantly inhibited the cleaved cas-12 expression (an ER-specific apoptotic factor) and alleviated cell apoptosis (27). This present study, we measured levels of cl cas-12 by the DOX treatment to indicate the apoptosis induced by ER stress, and further to investigate the changes of XBP1s during the ER stress. The expression of XBP1s was increased and nuclear translocated when applied DOX to H9c2 within 3 h, indicating that the DOX-induced UPR and alleviated ER stress by increasing XBP1s expression in a short time. With the extension of induction time, the level of XBP1s decreased, which was consistent with the decrease of PAK2 expression level and indicated that lack of PAK2 affected the activation of IRE/XBP1 pathway. Functionally, XBP1s as the downstream factor of miR-194-5p and PAK2, protected from DOX-induced cardiotoxicity.

Our result was consistent with other studies, showing that the XBP1s exerts cardioprotective effect. However, the expression levels of XBP1s under DOX treatment remain controversial. In a similar study of DOX-induced cardiotoxicity, the expression of XBP1s was downregulated in both 15 mg/kg (i.p.) DOX injected Institute of Cancer Research (ICR) mouse heart tissue and DOX-treated cardiomyocytes (27). In a study using SD rats, there was no significant change of XBP1s expression level in rat heart tissue after a single injection of DOX at 20 mg/kg (i.p.) (47). In another study on the role of ER stress in regulating the DOX-induced cardiotoxicity, XBP1 expression was upregulated in the heart tissues of C57BL/6J mice with a single injection of 20 mg/kg DOX (i.p.) (48). Unfortunately, neither of the latter two studies was conducted *in vitro* experiments nor was the function of XBP1 explored. These contrary results may be partially due to species heterogeneity, the difference in the dosage of DOX, and the selected myocardial tissue sites. These results indicated that ER stress is involved in the complexity of pathological mechanism regulation, and different induction conditions and external factors may cause different degrees of damage.

Currently, studies on miR-194 in cardiovascular diseases have involved in its serum expressions and the association with cardiac function impairment, suggesting the potential of miR-194 as a circulating marker. MiRNAs attracted extensive attention as potential biomarkers because they have many advantages: high conserved between species (12), partial tissue specificity (49), and stability of expression in circulation (50). In addition, miRNAs can be detected using sensitive techniques such as quantitative real-time PCR and next-generation sequencing. Therefore, whether miR-194-5p expression is also upregulated in circulation during the DOX-induced cardiotoxicity, and whether this abnormal expression can be used as a biomarker of the DOX-induced myocardial injury, remains to be further explored.

## Conclusion

In the DOX-induced cardiotoxicity, the miR-194-5p expression level was upregulated, and inhibition of miR-194-5p expression significantly alleviated the DOX-induced cardiotoxicity *in vitro* and *in vivo*, suggesting that the upregulation of miR-194-5p may be the cause of DOX-induced cardiotoxicity. Mechanically, miR-194-5p directly targeted PAK2 inhibited its expression, and participated in the regulation of DOX-induced cardiomyocyte apoptosis by affecting ER stress. Overexpression of PAK2 or XBP1s partially eliminated miR-194-5p induced cardiomyocytes apoptosis. Our study first identified the regulatory role of miR-194-5p/PAK2/XBP1s axis in DOX-induced cardiotoxicity, which provides a potential target for the prevention or treatment of the DOX-induced cardiotoxicity in its clinical applications.

## Data Availability Statement

The raw data supporting the conclusions of this article will be made available by the authors, without undue reservation.

## Ethics Statement

The animal study was reviewed and approved by Ethics Committee Medical College of Qingdao University.

## Author Contributions

HF and JW conceived and designed the study, and drafted the manuscript. HF, DX, and LD conducted most of the *in vitro* experiments and data analysis. WC, LY, and YW conducted the *in vivo* study. MW participated in collecting data. All authors reviewed and approved the manuscript.

## Conflict of Interest

The authors declare that the research was conducted in the absence of any commercial or financial relationships that could be construed as a potential conflict of interest.

## Publisher’s Note

All claims expressed in this article are solely those of the authors and do not necessarily represent those of their affiliated organizations, or those of the publisher, the editors and the reviewers. Any product that may be evaluated in this article, or claim that may be made by its manufacturer, is not guaranteed or endorsed by the publisher.
